# Supplementary material for: Content-rich biological network constructed by mining PubMed abstracts
Source: BMC Bioinformatics. 2004 Oct 8;5:147. doi: 10.1186/1471-2105-5-147 (PMC528731; doi:10.1186/1471-2105-5-147)
Supplement: Additional File 2 — The original results of the above study (non-essential files are deleted to keep the file size under the limit set by BMC bioinformatics). [file 1471-2105-5-147-S2.bz2 › chilibotAdditionalFile2/dip20/197ID8652557E680/html/ERCC3_GTF2H1.html]

 


 **ERCC3** and **GTF2H1** 
  
Found 427 abstracts in PubMed, retrieved 20.  
 

 What does Google say? 
 PDF only 
| .edu only 

---

**Interactive relationship** (e.g. stimulation, inhibition, etc)

**Neutral relationship**- We established that the carboxy terminal 125 amino acids are dispensable for both HR23B and damaged DNA binding, while interactions with transcription factor IIH TFIIH  [ **GTF2H1** ]   [ **ERCC3** ]  are significantly impaired by truncation of this domain.  Ref: 12509233 DNA Repair Amst , 2002
- Using computer analysis we identified several common motifs in the p62 proteins from different organisms, suggesting that these motifs could be involved in possible protein protein interactions within the TFIIH  [ **GTF2H1** ]   [ **ERCC3** ]  complex or with other transcription and DNA repair factors.  Ref: 12509240 DNA Repair Amst , 2002
- Analyses of AR mediated transcription suggest that the orchestrated interaction of AR with the transcription factors IIF TFIIF and IIH TFIIH  [ **GTF2H1** ]   [ **ERCC3** ] , and positive transcription elongation factor b P TEFb, may increase efficiency of transcriptional elongation from the androgen target genes, such as prostate specific antigen PSA.  Ref: 12648523 J Steroid Biochem Mol Biol, 2003
- Recent advances have shed some light on the structure of these factors, how conformational changes and intramolecular interactions regulate activity, and have revealed an expanded role for TFIIH  [ **GTF2H1** ]   [ **ERCC3** ]  in nuclear transcription.  Ref: 12672487 Curr Opin Genet Dev, 2003
- Moreover, when XPD mutations prevent interaction with the p44 subunit of TFIIH  [ **GTF2H1** ]   [ **ERCC3** ] , transactivation directed by certain nuclear receptors is inhibited, regardless of TTD versus XP phenotype, thus explaining the overlapping symptoms.  Ref: 12820975 Mol Cell, 2003
- General transcription factor IIH TFIIH  [ **GTF2H1** ]   [ **ERCC3** ]  consists of nine subunits cyclin dependent kinase 7 Cdk7, cyclin H and MAT1 forming the Cdk activating kinase or CAK complex, the two helicases Xpb  [ **ERCC3** ]  Hay and Xpd, and p34, p44, p52 and p62 refs 1 3.  Ref: 12853965 Nature, 2003
- Thus, TFIIH  [ **GTF2H1** ]   [ **ERCC3** ]  appears to protect promoters from PC4 mediated repression by relieving the topological constraint imposed by PC4 through the  **ERCC3**  helicase activity rather than by reducing the repressive activity of PC4 via its phosphorylation.  Ref: 12590132 J Biol Chem, 2003

**Non-interactive relationship** (e.g. studied together, co-existance, homology, etc.)

- Together with recent reports linking TFIIH  [ **GTF2H1** ]   [ **ERCC3** ]  in regulating NR function, our findings provide new data to further support an important link between DNA repair proteins and nuclear receptor function.  Ref: 12874288 J Biol ChemJ Biol Chem, 2003
- Mutations in some components of TFIIH  [ **GTF2H1** ]   [ **ERCC3** ]  are associated with three hereditary human syndromes xeroderma pigmentosum XP, Cockayne syndrome CS and trichothiodystrophy TTD.  Ref: 12509240 DNA Repair Amst , 2002
- Set1 association depends upon the TFIIH  [ **GTF2H1** ]   [ **ERCC3** ]  associated kinase that phosphorylates the Pol II C terminal domain CTD and mediates the transition between initiation and elongation, and Set1 interacts with the form of Pol II whose CTD is phosphorylated at serine 5 but not serine 2.  Ref: 12667453 Mol Cell, 2003
- Using recombinant TFIIH  [ **GTF2H1** ]   [ **ERCC3** ] , we have analyzed the role of TFIIH  [ **GTF2H1** ]   [ **ERCC3** ]  for alleviating PC4 mediated transcriptional repression and determined that the excision repair cross complementing  **ERCC3**  helicase activity of TFIIH  [ **GTF2H1** ]   [ **ERCC3** ]  is the enzymatic activity that alleviates PC4 mediated repression via beta gamma bond hydrolysis of ATP.  Ref: 12590132 J Biol Chem, 2003
- The results also showed that dispersal of recruited XPC and TFIIH  [ **GTF2H1** ]   [ **ERCC3** ]  from DNA damage SNS occurs within a short period after DNA damage.  Ref: 12713809 DNA Repair Amst , 2003
- Mutations in these residues also affected the ability of hTFIIE beta to stimulate TFIIH  [ **GTF2H1** ]   [ **ERCC3** ]  mediated phosphorylation of the carboxy terminal heptapeptide repeats of the largest subunit of Pol II.  Ref: 12665589 Mol Cell Biol, 2003
- By using a chromatin immunoprecipitation technique, we found that TFIIE is necessary for stable binding of TFIIH  [ **GTF2H1** ]   [ **ERCC3** ]  and RNAPII to the ADH1 promoter, whose activation requires TFIIE.  Ref: 12558798 Genes Cells, 2003
- Molecular characterization and developmental expression of the TFIIH  [ **GTF2H1** ]   [ **ERCC3** ]  factor p62 gene from Drosophila melanogaster effects on the UV light sensitivity of a p62 mutant fly.  Ref: 12509240 DNA Repair Amst , 2002
- Tumor suppressor p53 dependent recruitment of nucleotide excision repair factors XPC and TFIIH  [ **GTF2H1** ]   [ **ERCC3** ]  to DNA damage.  Ref: 12713809 DNA Repair Amst , 2003
- The p62 protein is a structural component of the TFIIH  [ **GTF2H1** ]   [ **ERCC3** ]  core and no syndromes have been identified up to date by mutations in this human gene.  Ref: 12509240 DNA Repair Amst , 2002
- In this study, the regulatory effect of p53, on the spaciotemporal recruitment of XPC and TFIIH  [ **GTF2H1** ]   [ **ERCC3** ]  to DNA damage sites, was investigated in repair proficient and deficient human cells in situ.  Ref: 12713809 DNA Repair Amst , 2003
- The archaeal transcriptional machinery is polII like but does not require ATP or TFIIH  [ **GTF2H1** ]   [ **ERCC3** ]  for open complex formation.  Ref: 12783891 J Biol Chem< MedlineTA>J Biol Chem, 2003
- Taken together, the results demonstrated that p53 plays a pronounced role in the damage recognition and subsequent assembly of repair machinery during GGR and the recruitment of XPC and TFIIH  [ **GTF2H1** ]   [ **ERCC3** ]  to CPD is p53 dependent.  Ref: 12713809 DNA Repair Amst , 2003
- Furthermore, these mutations conspicuously augmented the binding of hTFIIE beta to the p44 subunit of TFIIH  [ **GTF2H1** ]   [ **ERCC3** ] .  Ref: 12665589 Mol Cell Biol, 2003
- Previous studies showed that the repressive activity of PC4 on promoter driven transcription is alleviated by transcription factor TFIIH  [ **GTF2H1** ]   [ **ERCC3** ] , possibly through one of its enzymatic activities.  Ref: 12590132 J Biol Chem, 2003
- Colocalization experiments of the NER factors showed that in normal human cells, XPC and TFIIH  [ **GTF2H1** ]   [ **ERCC3** ]  are rapidly and efficiently recruited to DNA damage within SNS.  Ref: 12713809 DNA Repair Amst , 2003
- Often, phosphorylation of NRs by kinases that are associated with general transcription factors e.g. cdk7 within TFIIH  [ **GTF2H1** ]   [ **ERCC3** ] , or activated in response to a variety of signals MAPKs, Akt, PKA, PKC, facilitates the recruitment of coactivators or of components of the transcription machinery and, therefore, cooperates with the ligand to enhance transcription activation.  Ref: 12618210 Cell Signal, 2003
- Analysis of the results concentrated on the ring finger domain as the classic zinc finger domains were too constrained to provide much topological variety, whilst the TFIIH  [ **GTF2H1** ]   [ **ERCC3** ]  domain which has large unstructured loops did not behave well.  Ref: 12527307 J Mol Biol, 2003
- The antibody study indicated that they thus altered the conformation of one side of TFIIH  [ **GTF2H1** ]   [ **ERCC3** ] , consisting of p44, XPD, and Cdk activating kinase subunits, that is essential for the transition stage.  Ref: 12665589 Mol Cell Biol, 2003
- As the kinase subunit of TFIIH  [ **GTF2H1** ]   [ **ERCC3** ] , Cdk7 participates in basal transcription by phosphorylating the carboxy terminal domain of the largest subunit of RNA polymerase II.  Ref: 12853965 Nature, 2003
- We also show that TFIIH  [ **GTF2H1** ]   [ **ERCC3** ]  from TTD patients, but not from XP patients, exhibits a significant in vitro basal transcription defect in addition to a reduced intracellular concentration.  Ref: 12820975 Mol Cell, 2003
- Alleviation of PC4 mediated Transcriptional Repression by the  **ERCC3**  Helicase Activity of General Transcription Factor TFIIH  [ **GTF2H1** ]   [ **ERCC3** ] .  Ref: 12590132 J Biol Chem, 2003
- Together, AR may be able to interact with TFIIH  [ **GTF2H1** ]   [ **ERCC3** ] , P TEFb, and RPB2 to enhance transcription from AR target genes, such as prostate specific antigen that may play important roles in the prostate cancer progression.  Ref: 12593864 Biochem Biophys Res Commun, 2003
- Core NER enzymes XPA, XPF, XPG, and TFIIH  [ **GTF2H1** ]   [ **ERCC3** ]  are essential for recovery.  Ref: 12531012 DNA Repair Amst , 2002
- These results suggest that following initial damage recognition, the carboxy terminus of XPC may be essential for the recruitment of TFIIH  [ **GTF2H1** ]   [ **ERCC3** ] ,.  Ref: 12509233 DNA Repair Amst , 2002
- MAT1 is an assembly targeting factor for cyclin dependent kinase activating kinase CAK, which has been shown to functionally interact with general transcriptional factor TFIIH  [ **GTF2H1** ]   [ **ERCC3** ] , a known inducer of ER transactivation.  Ref: 12527756 J Biol Chem, 2003
- By contrast, recruitment of both XPC and TFIIH  [ **GTF2H1** ]   [ **ERCC3** ]  to DNA damage in SNS occurred much less efficiently in p53 Null or p53 compromised cells.  Ref: 12713809 DNA Repair Amst , 2003
- Here we show that the Drosophila TFIIH  [ **GTF2H1** ]   [ **ERCC3** ]  component Xpd negatively regulates the cell cycle function of Cdk7, the CAK activity.  Ref: 12853965 Nature, 2003
- Our previous reports demonstrated that AR interacted with TFIIH  [ **GTF2H1** ]   [ **ERCC3** ]  and positive transcription elongation factor b P TEFb,.  Ref: 12593864 Biochem Biophys Res Commun, 2003
- However, binding of TFIIH  [ **GTF2H1** ]   [ **ERCC3** ]  and RNAPII to CUP1 upon its activation did not require TFIIE.  Ref: 12558798 Genes Cells, 2003
- The preferred substrate for SCP1 is RNAP II phosphorylated by TFIIH  [ **GTF2H1** ]   [ **ERCC3** ] .  Ref: 12721286 J Biol Chem< MedlineTA>J Biol Chem, 2003
- TFIIH  [ **GTF2H1** ]   [ **ERCC3** ]  is a multiprotein complex that has a central role in the RNA pol II mediated transcription, in DNA repair and in the control of the cell cycle.  Ref: 12509240 DNA Repair Amst , 2002
- We also show that, as complexed within TFIIH  [ **GTF2H1** ]   [ **ERCC3** ] , the cyclin dependent kinase 7 kinase does not possess the activity to phosphorylate PC4.  Ref: 12590132 J Biol Chem, 2003
- Expression of several nuclear genes involved in cell signaling, cell architecture, energy metabolism, cell growth, apoptosis including general transcription factor TFIIH  [ **GTF2H1** ]   [ **ERCC3** ] , v maf, AML1, was induced in Rho0 cells.  Ref: 12495447 Mol Cancer< MedlineTA>Mol Cancer, 2003
